# Relevance of HTLV-1 proviral load in asymptomatic and symptomatic patients living in endemic and non-endemic areas of Argentina

**DOI:** 10.1371/journal.pone.0225596

**Published:** 2019-11-22

**Authors:** María Verónica Pineda, María Belén Bouzas, Mirta Remesar, Ariel Fridman, Carlos Remondegui, Lilia Mammana, Natalia Altamirano, Patricia Paradiso, Patricia Costantini, Luciana Tadey, Paula Aulicino, Andrea Mangano

**Affiliations:** 1 Laboratorio de Biología Celular y Retrovirus-CONICET, Unidad de Virología y Epidemiología Molecular, Hospital de Pediatría "Prof. Dr. Juan P. Garrahan", Ciudad Autónoma de Buenos Aires, Argentina; 2 Unidad de Virología, Hospital de Infecciosas “Francisco J. Muñiz”, Ciudad Autónoma de Buenos Aires, Argentina; 3 División de Análisis Clínicos, Hospital de Infecciosas “Francisco J. Muñiz”, Ciudad Autónoma de Buenos Aires, Argentina; 4 Centro Regional de Hemoterapia Garrahan, Hospital de Pediatría "Prof. Dr. Juan P. Garrahan", Ciudad Autónoma de Buenos Aires, Argentina; 5 Laboratorio Central de Jujuy, Jujuy, Argentina; 6 Servicio de Infectología y Enfermedades Tropicales, Hospital San Roque, Jujuy, Argentina; 7 Servicio de Hemoterapia, Hospital Durand, Ciudad Autónoma de Buenos Aires, Argentina; 8 Instituto de Oncología Ángel H. Roffo, Ciudad Autónoma de Buenos Aires, Argentina; Hospital Clinico Universitario San Carlos, SPAIN

## Abstract

HTLV-1 proviral load (pVL) in peripheral blood mononuclear cell (PBMCs) is proposed as a marker of disease progression but its role still remains controversial. The aim of this study was to evaluate the levels of HTLV-1 pVL in symptomatic patients and asymptomatic HTLV-1 carriers. In this cross-sectional study the pVL was measured by Real Time PCR in 102 asymptomatic carriers and 22 symptomatic patients (5ATLL, 15 TSP and 2 uveitis). We observed that the HTLV-1 pVL was significantly higher in symptomatic patients (median = 4.99 log10 HTLV-1 copies /106 PBMCs) compared to asymptomatic HTLV-1 carriers (median = 4.38 log_10_ HTLV-1 copies /10^6^ PBMCs; p = 0.0030). A wide variation on the HTLV-1 pVL levels among asymptomatic HTLV-1 carriers was observed with some pVL as high as those observed in symptomatic patients. The asymptomatic HTLV-1 carriers were divided according to the place of birth and the highest levels of pVL were detected among patients from endemics areas from the North of Argentina. Our results reinforce the usefulness of the proviral load would be a prognostic marker of HTLV-1 disease progression. Moreover, host, viral or socio-environmental factors cannot be excluded as determinant of high proviral load.

## Introduction

HTLV-1 is the etiologic agent of adult T-cell leukemia/lymphoma (ATLL) [1]^,^ [2] and is also related with HTLV-1-associated myelopathy/tropical spastic paraparesis (HAM/TSP)[3]^,^ [4] and other inflammatory diseases such as uveitis [5], dermatitis, and arthritis[6], demonstrating a broad spectrum of systemic inflammation caused by the viral infection. The majority of HTLV-1 patients remain asymptomatic throughout their lives, while 2.5–5% developed ATLL [7]^,^ [8] and 0.3–2% HAM/TSP [9]^,^ [10]. About 10 to 20 million people worldwide are infected with HTLV-1 with foci of endemicity in South Japan, the Caribbean, Sub-Saharan Africa and in several areas in South America including: Argentina, Bolivia, Brazil, Colombia, French Guyana, Paraguay, Peru, and Uruguay [11]^,^ [12]. In Argentina, HTLV-1 infection has been documented in several regions, particularly in the northeast provinces neighboring to Bolivia, Paraguay and Chile. The highest prevalence of the infection has been observed in the northern provinces of Jujuy, Salta and Tucuman reaching levels of 1% or higher in some Blood Banks [13]. In contrast, in the center region of the country including the provinces of Buenos Aires, Córdoba and Santa Fe the reported prevalence from blood donors is around 0.02–0.04% [14]^,^ [15].

HTLV-1 produces only small amounts of extracellular virions and the majority of the virus remains integrated in the DNA of T CD4^+^ cells [16]. The viral spread is mainly through clonal expansion of infected cells [17]^,^ [18] or the transfer of viral RNA via cell-cell contact known as the virological synapse. RNA is almost undetectable in human serum and therefore, viral burden is quantified as the proportion of peripheral blood mononuclear cells (PBMCs) that carries integrated HTLV-1 provirus, referred as proviral load (pVL). Previous studies in HTLV-1 pathogenesis have focused on pVL in PBMCs as a marker of disease progression[19]^,^ [20]^,^ [21]^,^ [22]. These studies suggest that high pVL is related with high risk of developing HTLV-1 associated HAM/TSP and ATLL, but these results still remain controversial and few studies addressed the question regarding the threshold pVL value[23]. Demontis et al, suggests that this value could be between 10–15% HTLV copies/ 100 PBMC [21]. The aim of this study was to evaluate the levels of HTLV-1 pVL in asymptomatic and symptomatic patients born in different regions of Argentina. The results showed that the pVL plays an important role in the pathogenesis of the HTLV-1 associated diseases and as a biomarker in the clinical follow up.

## Materials and methods

### Study group

This cross-sectional study was conducted on 124 HTLV-1 infected subjects including i) 13 blood donors and 3 Hematopoietic Cells Umbilical Cordon Blood donors from the Regional center of Hemotherapy Garrahan, Hospital de Pediatría “Prof. Dr. Juan P. Garrahan”, Buenos Aires; ii) 41 adult patients from different Hospitals from Buenos Aires, and iii) 67 adult patients from the Infectious Diseases and Tropical Medicine Service of the Hospital San Roque, San Salvador de Jujuy-Jujuy (north region of Argentina). Symptomatic patients included 5 individuals with ATLL, 15 with HAM / TSP and 2 with uveitis; none of them presented any other disease besides the detailed ones. The main sources of asymptomatic carriers (AC) were blood donors.

The diagnosis of HTLV-1 was performed by serological screening tests and confirmatory Western Blot test at each clinical setting. Seroindeterminate samples were studied by HTLV-1 and HTLV-2 specific PCR to define HTLV status at the "Laboratorio de Biología Celular y Retrovirus, Hospital de Pediatría Prof. Dr. Juan P. Garrahan".

The study was approved by Institutional Review board and The Ethics Committee of the Hospital de Pediatría "Prof. Dr. Juan P. Garrahan", (Protocol N° 639). Written informed consent was obtained from all individuals.

### Clinical samples

Clinical samples were collected between 2001 to 2013. PBMCs from all patients were isolated from EDTA blood samples by Fycoll-Hypaque® density gradient centrifugation and washed twice in phosphate buffered saline. Dry pellets containing 2 x 106 or 5 x 106 cells were stored and kept at -20°C until DNA extraction. Genomic DNA from PBMCs was extracted using the purification kit QIAmp DNA Mini Kit (QIAGEN®) according to the manufacturer’s recommendations. Once the DNA was extracted the HTLV-1 pVL was measured.

### Proviral load quantification of HTLV-1

HTLV-1 pVL in PBMCs was measured by an in house real-time quantitative PCR (qPCR) assay using SYBR Green as we previously described [24]. Briefly, primers targeting the pol region were standardized against the MT2 cell line and HTLV-1 copy number was normalized to the amount of cellular DNA by quantitation of the albumin gene. The assay had a detection limit of 400 HTLV-1 copies/106 PBMCs, with a broad range of quantitation (2.6 log10 to >5 log10), without cross-reactivity with HTLV-II or HIV-1 and with an intra-sample CV% less than 5%[24]. In order to verify the reproducibility of our qPCR, we performed external quality control using clinical samples previously tested.

### Statistical analysis

Standard descriptive statistics were used to describe the baseline characteristics of the population. Results were expressed as median and interquartile range (IQR). Both the Mann-Whitney U test or the Kruskal-Wallis test were used to compare continuous data between AC and symptomatic patients. Chi-square test was used for categorical data. A two-sided P-value of less than 0.05 was considered to indicate statistical significance. Data were analyzed using Graph Pad Prism® 5 software package 4.91.

## Results

A total of 124 individuals infected with HTLV-1 were included in this study. Patients were divided into two groups according to the presence or absence of symptoms. Of them, 102 were classified as asymptomatic HTLV-1 carriers being the main source blood donors and 22 as symptomatic patients.

Demographic characteristics of the patients are detailed in Table 1. In the group of AC, 63% were women and 37% were men and among the symptomatic patients, 68% were women and 32% were men. No significant difference was observed in the gender distribution between both groups. According to the age at enrollment, symptomatic patients were significantly older than AC with a median of 53 years (IQR = 39–56) and 35 years (IQR = 30–49), respectively (p = 0.0107). The majority of AC (60%) and symptomatic patients (77%) were born in the North provinces of Argentina considered an endemic region (Chaco, Corrientes, Jujuy, Misiones, Salta and Tucuman). A total of 20 patients were born outside Argentina including individuals from Bolivia, Peru, Paraguay, Chile and Korea. Of them, 19 were AC and only 1 symptomatic patient with HAM/TSP. A region classification was established in order to separate subjects born in endemic and non-endemic areas. The North region corresponds to the endemic area of Argentina, and was subdivided in the Northwest (Jujuy, Salta and Tucuman) and the Northeast areas (Chaco, Corrientes and Misiones). The Center region corresponds to a non-endemic area of Argentina, including Buenos Aires City, Buenos Aires Province, Santa Fe and San Juan. The countries were HTLV-1 is endemic such us Bolivia, Peru and Paraguay were grouped as “other countries”. Two asymptomatic HTLV-1 carriers, one from Chile and the other from Korea were excluded from this classification.

**Table 1:** Baseline characteristics of HTLV-1 infected patients.

	Asymptomatic carriers	Symtomatic patients	p
	(n = 102)	(n = 22)	
**Gender, Female/male**	64/38	15-Jul	0.0036
Age at enrollment, ys (IQR)*	35 (30-49)	53 (39-56)	0.0107
**Place of birth**			
**Aregntina**			
**Endemic Area**			
NE*	1	3	
NW**	60	14	
**Non endemic area**			
Center area	11	4	
**Other country**			
Bolivia	6		
Peru	6	1	
Paraguay	5		
Chile	1		
Korea	1		
**Unknown**	**11**	**0**	** **

*NE (North East)

** NW (North West)

**Fig 1:**
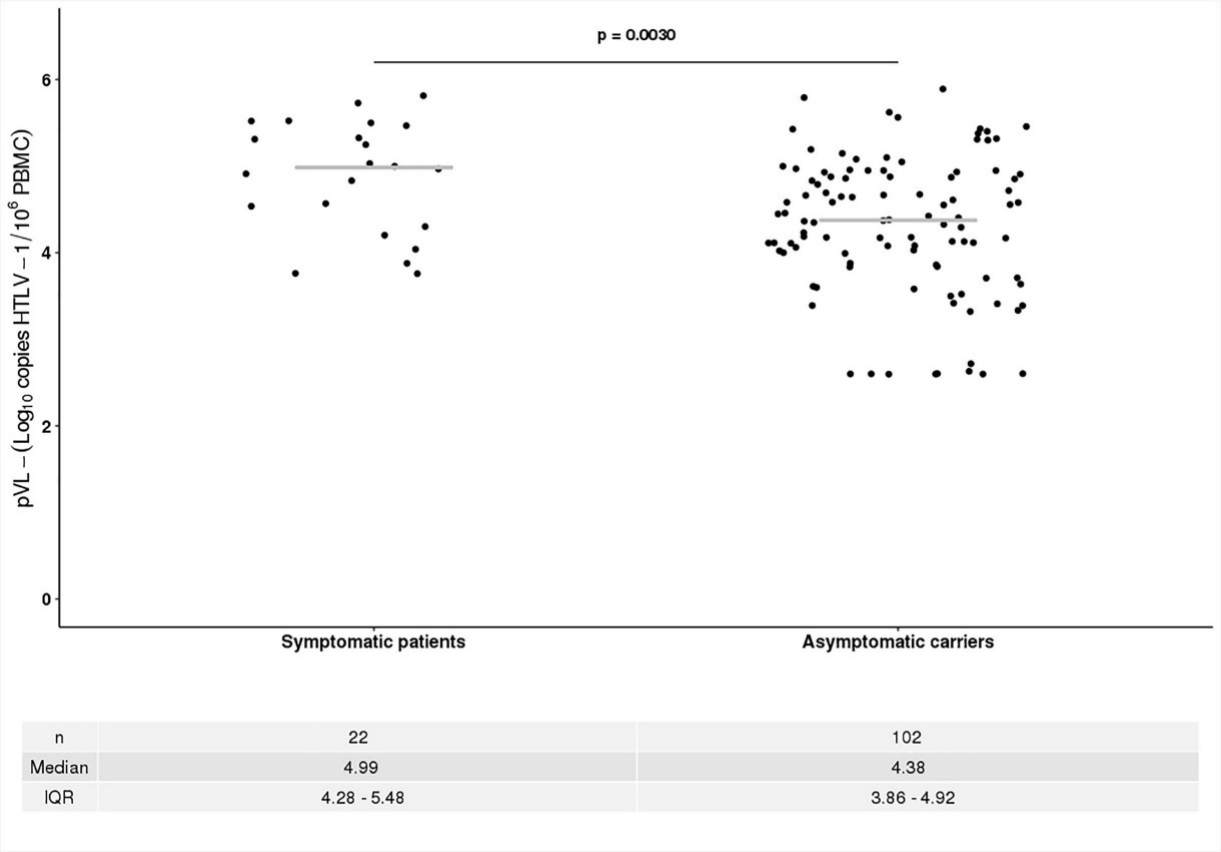
Proviral load of symptomatic carriers vs asymptomatic HTLV-1 carriers.

Quantification of pVL was assessed on PBMCs by real time qPCR. HTLV-1 pVL ranged from 2.6 log_10_ HTLV-1 copies /10^6^ PBMCs to 5.89 log_10_ HTLV-1 copies /10^6^ PBMCs. HTLV-1 pVL was significantly higher in symptomatic patients (median = 4.99 log_10_ HTLV-1 copies /10^6^ PBMCs–IQR = 4.28–5.48) compared to AC (median = 4.38 log_10_ HTLV-1 copies /10^6^ PBMCs, IQR = 3.86–4.92; p = 0.0030; Fig 1). The clinical spectrum of HTLV-1 associated diseases among symptomatic patients is described in Table 2. We analyzed if there was any difference between the levels of HTLV-1 pVL in symptomatic patients with different diseases. Of them 15 patients had HAM/TSP, 5 had ATLL and 2 had uveitis. We found that HTLV-1 pVL among patients with HAM/TSP were similar to those with ATLL (5.03 log10 HTLV-1 copies /10^6^ PBMCs and 4.83 log_10_ HTLV-1 copies /10^6^ PBMCs; respectively). Regarding to gender differences we observed that women were more prone to develop HAM/TSP, while male patients mostly developed ATLL (p = 0.0139). Furthermore, the number of patients with uveitis was too low so they were not included in the comparison.

**Table 2:** HTLV-1 proviral load of symptomatic patients.

Type of disease	Total	Gender Female/Male	Region of birth	pVL, Log_10_copies/10^6^ PBMCs	pVL as % of DNA copies/100 PBMCs
			North / Center regions	Median (IQR)	
ATLL	5	1/4	2/3	4,83 (3.82 – 5.41)	6.7 (0.66 – 25.70)
HAM/TSP	15	13/2	14/1	5.03 (4.57 – 5.50)	10.76 (3.72 – 31.62)
Uveitis	2	1/1	2/0	3.90 (3.76 – 4.04)	0.79 (0.58 – 1.10)

**Fig 2:**
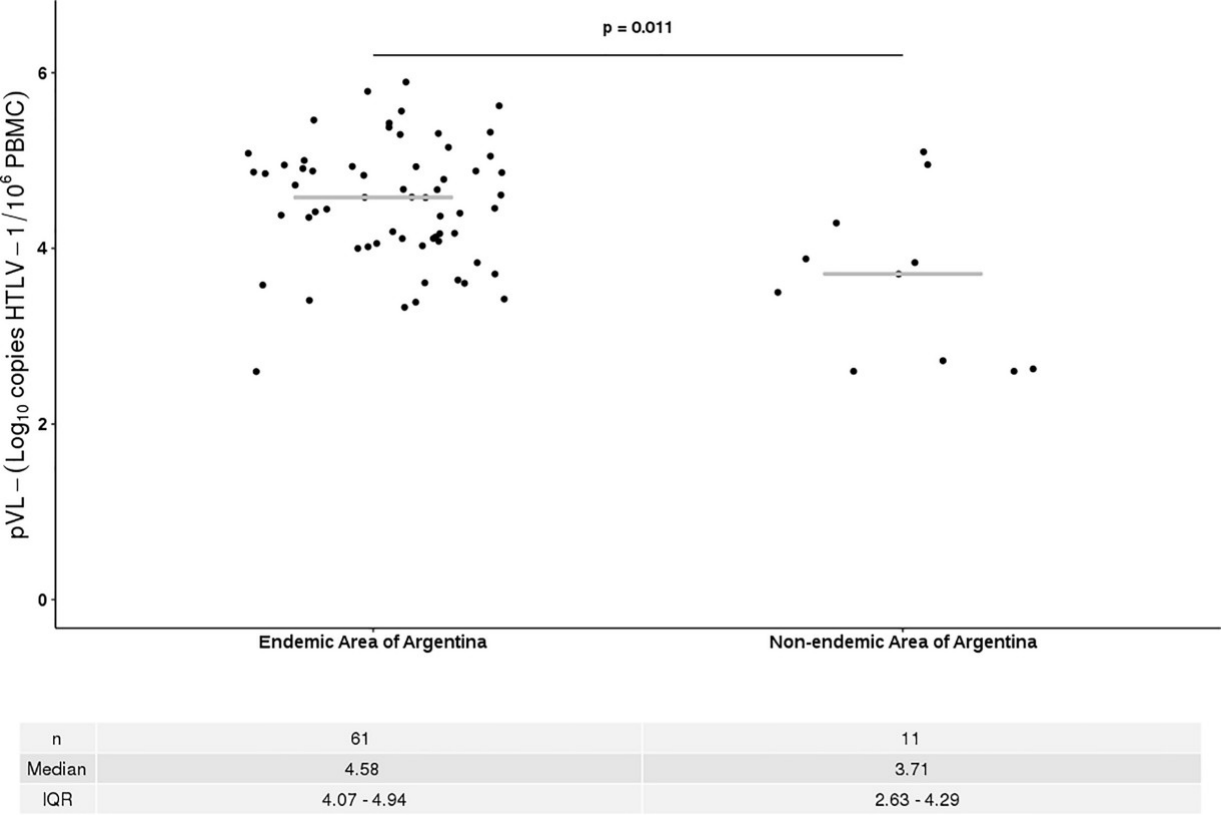
Proviral load of asymptomatic HTLV-1 carries from the North region vs Center region.

A wide variation on the HTLV-1 pVL was observed among AC. In some of them, HTLV-1 pVL was as high as those observed in symptomatic patients. Given that the majority of symptomatic patients were born in endemic area, the place of birth was evaluated as a possible factor that may be related to the level of the pVL from AC. Therefore, AC were divided according to the place of birth in those from endemic, non-endemic areas of Argentina and “others countries”. Of them, 61 were born in an endemic area and 11 in a non-endemic area of Argentina and 17 in “others countries”. We found that AC from the endemic area of Argentina had significantly higher HTLV-1 pVL (median = 4.58 log_10_ HTLV-1 copies/10^6^ PBMCs; IQR = 4.07–4.94) compared to AC from the non-endemic area (median = 3,71 log_10_ HTLV-1 copies/10^6^ PBMCs; IQR = 2.63–4.29; p = 0.011; Fig 2). In contrast, no differences were observed in the pVL of patients from "other countries" (median = 4.23 log10 HTLV-1 copies/106 PBMCs; IQR = 3.86–4.65); and those from the endemic and non-endemic area of Argentina. Finally, it is important to remark that the reproducibility of our real time PCR through the external quality control showed a variation less than the total error value accepted for the method (0.5log).

## Discussion

There is no available biomarker to predict the risk of progression from asymptomatic to symptomatic status in HTLV-1 infected subjects. However, HTLV-1 pVL has been widely used as a surrogate marker of disease progression and high levels have been associated with a symptomatic status of the disease. Previous publication defined three different cut off of pVL [21], which are expressed in percentage of HTLV-1 DNA copies/ 100 PBMCs, being low (<1% ≅ <Log_10_ 4), medium (1–10% ≅ Log_10_ 4—Log_10_ 5) and high (> 10% ≅ > Log_10_ 5) respectively.

As has been previously reported by several groups, in our study group, symptomatic patients had higher pVL compared to the asymptomatic patients. [21]^,^ [8]^,^ [25]^,^ [26]. Among patients with HAM/TSP (n = 15), which represent 68% of the entire symptomatic group, 47% of them had medium pVL levels, while 53% had high pVL. Moreover, when patients with HAM / TSP were classified according to the OSAME score [4], no difference was observed between their pVL and their clinical stage. Among patients with ATLL, 3 were diagnosed as lymphoma and two as leukemia with a high variability in their pVL levels. The lymphomas showed low and high pVL values, while in patients with leukemia we found high, medium and low pVL. This high variability may be due to the differences in the clinical stages of the disease. Eventhough, the number of cases with ATLL in our study is low in order to evaluate whether there is a trend in the pVL levels.

In contrast, among the asymptomatic patients, 27% of them had low pVL and 56% had medium pVL levels, still 17% of the patients in this group showed high values similar to those detected among the symptomatic patients. These results suggest that asymptomatic patients with a high level of pVL (> 10% of HTLV-1 DNA copies/ 100 PBMCs, or ≥ 5 log_10_ copies / 10^6^ CMT) are in a high risk for the development of the disease. We consider this finding reinforces the idea that the quantification of the proviral load would be a prognostic marker of disease progression. For all these reasons, we consider that medical and laboratory follow-up by pVL may provide early information of clinical value in the progression from asymptomatic to symptomatic stage; as well as for detecting and treating the early manifestations the HTLV-1 associated disease. In this sense, other studies have revealed that pVL in asymptomatic patients remained stable over time with slight fluctuations [27]^,^ [22]. In longitudinal studies the switch from the asymptomatic to symptomatic stage is difficult to predict since HAM/TSP and ATLL occurs after a long period of clinical latency (10–40 years). However, results from a large cohort during a follow up study [8] found that 14 out of 1,259 asymptomatic patients which developed ATLL, their pVL at baseline were higher and increased over time when they were compared to those that remained asymptomatic. These studies support the hypothesis that high pVL or major individual variations are associated with the progression to a symptomatic stage.

In our study a higher proportion of HTLV-1 infected patients were women; this may be due to a higher male-to-female transmission efficiency during sexual intercourse. Also, we observed a higher number of patients with HAM/TSP than ATLL, and women were more likely to develop HAM/TSP while men tend to develop ATLL. Different viral or host factors may play an important role in the pathogenesis of HTLV-1 such as genetic background, local factors and immune response that may be important in the pathogenesis of HTLV-1.

Previous studies reported significant differences in the pVL levels according to the sex and age of the patients[8]. Our results showed no association between pVL levels and gender and age distribution, in concordance with other studies [23]^,^ [28]^,^ [29]. As we previously discussed, in our study we observed some asymptomatic patients with high pVL resembling those among symptomatic patients with similar age range, 40–50 years. We consider that these patients may be at a higher risk to develop disease, so it is important to offer a close clinical and laboratory follow up.

An interesting find was the differences in the pVL levels among the AC group according to the place of birth, where the highest levels of pVL were detected among patients from endemics areas of Argentina. This suggests that viral factors, host factors–such as ethnicity, HLA, co-morbidities, immune response—or socio-environmental factors may influence the pVL as well as the viral load population as has been extensively described for HIV [30]^,^ [31]. It is well known that vertical transmission, breast-feeding and sexual transmission are routes that contribute mainly to mantain the prevalence of HTLV-1 in endemic areas. In the situation where no therapeutic intervention has been done it is possible that the proviral load population in this area is higher in comparison with a non or low endemic areas[32][33][34][35].

The study has several limitations. This is a cross-sectional study therefore there was no chances to explore the predicted value of developing disease in carriers with high level of htlv-1 proviral load. Further follow-up of these patients and prospective investigations should provide data to support more detailed conclusions. AC with high proviral load were not re-evaluated after this study to see if they start with symptoms such as bladder dysfunction, arthritis, thyroid disease, etc. Asymptomatic carriers (AC) in the present study did not have any symptoms or any clinical condition associated with HTLV-I infection moreover the main source in this group were blood donors, who once the diagnosis was performed they received clinical evaluation.

In summary, our results indicate that the level of proviral load of HTLV-1 in PBMCs is associated with clinical disease, as HAM/TSP and ATLL. These levels remain low in asymptomatic carriers and high in symptomatic patients. Eventhough, asymptomatic patients from endemic areas in Argentina showed higher levels than those from non-endemic areas from our country. Our data support the hypothesis that HTLV-1 proviral load plays an important role in the pathogenesis of HAM / TSP and ATLL and as a biomarker in the clinical follow up. However, the presence of co-morbidities is another aspect to consider when evaluating the pVL; given that the presence of parasitic and viral co-infections may contribute to the clonal expansion of infected lymphocytes, leading this to an increase in the pVL levels. For these reasons, we consider that it is important to monitor the pVL levels over time and to evaluate the intra- and inter-individual variability. Further studies investigating other factors that might impact on proviral load levels may open new insights on HTLV pathogenesis.

## Supporting information

S1 STROBE Checklist (DOC)
